# Thermomechanical Assessment of Recovered PA12 Powders with Basalt Filler for Automotive Components

**DOI:** 10.3390/polym16192682

**Published:** 2024-09-24

**Authors:** Francesco Napolitano, Ilaria Papa, Francesca Cimino, Valentina Lopresto, Pietro Russo

**Affiliations:** 1 Institute of Polymers, Composites and Biomaterials, National Research Council, Via Campi Flegrei 34, 80078 Pozzuoli, Italy; francesca.cimino@ipcb.cnr.it (F.C.); pietro.russo@ipcb.cnr.it (P.R.); 2 Department of Chemical, Materials and Production Engineering, University of Naples “Federico II”, P.le Tecchio 80, 80125 Naples, Italy; ilaria.papa@unina.it (I.P.); valentina.lopresto@unina.it (V.L.)

**Keywords:** PA12, basalt, DMTA, biobased, thermomechanical analysis

## Abstract

Additive manufacturing processes allow for precise and efficient production, but it is estimated that one-third of the materials used results in waste. Further improvement in a sustainable perspective could come from the ability to manage these scraps and from the exploration of different routes for recovery and reuse. The Selective Laser Sintering process is particularly sensitive to this issue due to the waste ratio which can reach a very high quantity of not-sintered virgin powders. In this research study, recovered PA12 powders, preliminarily characterized through thermal and mechanical analysis, were mixed with 15% basalt powder to improve their aspect and thermomechanical resistance. The influence of basalt powder (BP) on mechanical properties as well as on the thermal stability of polyamide12 (PA12) powder composites was investigated. A study conducted on mechanical properties showed that polymeric composites’ stiffness and hardness were influenced by adding filler, thus improving mechanical parameters. On the other hand, the application of thermogravimetric analysis allowed us to determine the composite’s thermal stability. The objective is to obtain a recovered fully biobased material that could be used to substitute the petroleum-derived polymeric ones currently employed in the production of interiors and shells in the automotive sector.

## 1. Introduction

Currently, the use of renewable resources and the development of efficient recycling procedures are key efforts in the manufacturing sector. These efforts aim to reduce companies’ carbon footprints and lower their contributions to global warming. In this frame, thermoplastic biobased polymers have obtained wide applications due to their match with both requirements, and the sustainability of their use is particularly enhanced if they are transformed through additive manufacturing. Nevertheless, the choice to prefer 3D printing does not ensure clear advantages; as a matter of fact, it requires more energy consumption due to its slow production rate and produces an amount of waste which usually varies from 25% to 50% [1,2]. These scraps include, among others, supports, wasted filament in the case of machine misfunctions and used parts because of insufficient property or functionality [3]. Moreover, some 3D printing processes, especially those based on the use of powders, are set by printing producers to use virgin material batches for each new printing process. Among these, Selective Laser Sintering (SLS) [4] leads to the production of large quantities of unsintered polymeric powders. The quantity of the powder waste produced depends on the ratio between the volume of the workpiece and the total working volume of the SLS machine. Given that many of these powders also have a high market price, it is clear that there is a need to recover this waste to transform it into new valuable products [5,6,7].

In other words, constraints, related to environmental impact reduction and money saving, force us to investigate other methods of waste management. Among the most expensive polymeric not-sintered powders, there is polyamide 12: an interesting biobased thermoplastic polymer, widely used in SLS for medical device production [8]. Its sustainability is also coupled with its excellent mechanical and thermal behavior for highly durable components [9]. So, PA12 represents an excellent material for developing a recovery procedure to avoid its disposal [10]. The recovery of this material, generated by the not-sintered powders of laser-based additive processes, is a hot topic from the perspective of sustainability improvement. Early pioneeristic studies were carried out on the wastes provided by the additive manufacturing of metals. In these studies, a preliminary analysis of these powders was performed in order to verify their properties and the possible microstructural modifications induced by the interaction with laser beams [11,12]; the final aim of these studies was, indeed, to explore the possibility of their reuse in new additive manufacturing processes. As a matter of fact, successive studies were focused on the characterization of the workpieces obtained by 3D printing processes with recovered metal powders [13,14]. Contaldi et al. investigated an opportunity to use these powders, recovered from the SLM process, for other powder-based processes, such as direct metal sintering [15], widening the recycling and reuse routes. Regarding metal powders, the main reasons for improving their reuse are related to material production costs. All the examined alloys are high-performance alloys, which require a great quantity of energy spent for their production and thus for their recycling.

From the point of view of polymer production and recycling, the main issues are related to waste management, since they are considered materials with high environmental impact. Moreover, biobased polymers allow for a reduction in the use of non-renewable resources, and if they are also recyclable, both characteristics allow us to improve the sustainability of the products. Nevertheless, some of these biopolymers are still non-biodegradable, and some efforts have to be made to improve reuse procedures. Generally, the aim is to avoid expensive and energy-demanding production processes from biomass which usually require more investment to be conducted [16]. In light of these premises, it is desirable to preliminarily characterize the recovered polymer powders to evaluate them as raw materials to be used for other production processes, identify the most suitable manufacturing technology, and subsequently, proceed with the characterization of the workpieces, thus moving from microscalar to macroscalar characterization. 

Recently, some authors already explored the opportunity of using recovered powders. Among these, Gomes et al. attempted a further use of PA12 powder waste with the same SLS technology [17]; Yang et al. considered the optimization of SLS parameters based on powder degradation [18]; Magi et al. developed several mixtures for use in FDM [19]. However, despite the knowledge already acquired, the significant amount of waste and the particularly high cost of the reference raw material (PA12) mean that further investigations are necessary to better address this challenge. Furthermore, the intense focus on the reuse of PA12 for other 3D technologies does not allow us to take into consideration other technological processes that could represent the way to obtain competitive parts by solving many problems related to powder degradation. Among the sectors of use of PA12, noteworthy is the automotive one for the production of interiors and the design of unconventional shapes; however, it is largely widespread in its black-colored version, which is the most common commercial choice [20].

The objective of this research work is to evaluate the quality and properties of recovered and not-sintered PA12 powders to develop a new series of parts to be used in the automotive sector, replacing similar petroleum-based ones. Since the recovered powders are white and the expected qualitative mechanical characteristics, provided by the material’s technical data sheet, are worse than polypropylene and polyamide 6, we decided to use a proper filler which allows us to obtain an enhancement in mechanical properties, black coloration and no decrease in eco-compatibility. On these premises, interest was directed to basalt: a mineral filler obtained from lava rocks. The use of this filler as the reinforcement of polymeric matrices is widely reported in the literature with scientific evidence mainly focused on systems with basalt contents ranging from 5% to 40% in weight [21,22]. For this preliminary study, in order to avoid an excessive weight increase (the density of basalt is 2.8 g/cm^3^) and conglomerate formation, we decided to use 15% by weight of basalt filler. The samples, produced by compression molding, were characterized in terms of thermal tests, using DSC and TGA, and mechanical properties by quasi-static three-point bending tests and dynamic tests in dual-cantilever bending configuration, performed using DMTA. This approach is in line with the above-mentioned procedure, from the microscale to the macroscale; as a matter of fact, thermal characterization could give indications on the structural modifications, induced by laser beam interaction, which affect thermal properties such as melting point, glass transition temperature and crystallization temperature [23]. They could also give important information on the effects of basalt addition and its nucleating effects [24]. Mechanical tests are mandatory to assess the resistance and stiffness of the workpieces produced; by considering the macroscopical properties under operating conditions, the three-point bending test gives important information on the toughening effects of basalt addition. Finally, the dynamic mechanical thermal analysis is important for acquiring information on the structural behavior, under thermal changes, of the produced samples. These latter aspects are deeply affected by the manufacturing technology and the thermal profile adopted for compound and workpiece production [25].

## 2. Materials and Methods

### 2.1. Sample Preparation

The polyamide12 powders were recovered by a local company which uses, for its SLS process, SINTERIT PA12 Industrial FRESH. For basalt, the filler chosen was a commercial stimulant for leaves which has a powder shape, with a granulometry range of 15–50 µm, provided by FARINA DI BASALTO^®^ (Orvieto, Italy). The processing steps for sample production started from the mixing of the melted polymer and the filler, for creating the compound; this action was carried out in a Brabender Plastograph EC twin-screw compounder (Anton Paar GmbH, Graz, Austria), setting a temperature of 200 °C, a screw speed of 60 rpm and a residence time of 5 min. After this, the mixed polymer was processed in a COLLIN P400E one-column platen press (COLLIN Lab & Pilot Solutions GmbH, Maitenbeth, Germany), by setting the temperature to 200 °C and applying the following pressure cycle: 2 min at 0 bar, 1 min at 5 bar, 1 min at 10 bar and 1 min at 20 bar. After cooling, plates of 2 mm thickness were obtained.

In order to evaluate the properties of the recovered PA12 powders, the effects of the processes and the effects of basalt filler addition, three types of samples were produced. The first type, named PA12_raw, was produced directly by pressing the recovered polyamide powders; the second type, identified with PA12_mix, was firstly processed by mixing the recovered powders in the compounder and then pressing the obtained polymer without filler; the third type, called PA12_bas, was obtained by mixing the recovered PA12 powders and basalt in the compounder and then performing the pressing procedure. The main reason for producing three types of samples is due to the need to split the effects of each processing step. By using PA12_raw, it is possible to evaluate the effect of laser interaction during the SLS process; by using PA12_mix, the influence of the mixing procedure was evaluated, performed at melting temperature; finally, PA12_bas allows us to investigate the contribution of basalt powder addition.

### 2.2. Characterization

The produced samples were subjected to thermal and mechanical characterization. From a thermal point of view, a Differential Scattering Calorimetry analysis was conducted using DSC TA Q2000 (TA Instruments New Castle, DE, USA) with two heating phases at a rate of 10 °C/min, from 25 °C to 250 °C, separated by a cooling phase from 250 °C to 25 °C at the same thermal scan rate. The thermogravimetric analysis was performed on Perkin Elmer Diamond Pyris from 25 °C to 800 °C using a 10 °C/min heating ramp and nitrogen atmosphere. Dynamic thermomechanical characterization was carried out with a Perkin Elmer Pyris Diamond DMA instrument (PerkinElmer, Shelton, CT, USA) on a temperature range from −100 °C to 150 °C using a 3 °C/min heating ramp and a dual-cantilever bending configuration. Finally, the sample with dimensions 12.7 × 80 × 2 mm was subjected to a 3-point bending test, using an Instron 4505 general-purpose testing machine (Instron Corporation, Canton, MA, USA), equipped with a 1kN load cell, setting a crosshead speed of 2 mm/min and an opening of 50 mm, as required by the ASTM D790 standard method [26].

## 3. Results and Discussion

### 3.1. Thermal Characterization

**Figure 1:**
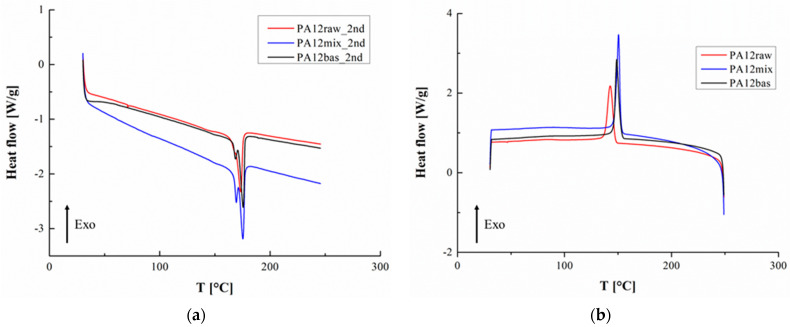
(**a**) Representative curves of the second heating run in the DSC analysis, (**b**) representative curves of the cooling run.

Figure 1a,b show the thermograms relating to the second heating ramp and the cooling phase, respectively, while Table 1 contains some calorimetric parameters obtained from their processing. For the considered sample, the melting signal appears to be split into two signals due to morphological–structural aspects also triggered by the laser sintering process [27]. The recovered PA12 has, in the first heating run, a melting temperature which is very close to that of the virgin material; starting from the second heating run, this temperature shows an initial decrease of 10 °C, which could reach 17 °C in the case of the third melting step. Therefore, the recovered PA12 raw material retains the characteristics of the virgin material, but a further mixing process and compounding with basalt powder induce a decrease in this thermal parameter due to structural modifications undergone by the reference biobased polymer matrix. According to Dorigato and Fambri, it is possible to estimate the degree of crystallization of the examined samples using the following formula [28]:

(1) $$\chi =100\frac{\Delta H_{i}}{\Delta H_{PA}\times f}$$

where $\Delta H_{i}$ is the crystallization enthalpies evaluated from DSC thermograms, $\Delta H_{PA}$ is the reference heat of the fusion of fully crystalline PA12, taken as 95 J/g [29], and f is the matrix weight fraction. Evidently, an increase in the degree of crystallinity is noted following both the processing of the pure matrix and the inclusion of the filler which can probably favor heterogeneous nucleation phenomena (see Table 1).

The TGA analysis does not allow us to distinguish the samples examined in terms of thermal stability, showing almost comparable derived signals (DTG peak) due to the same thermal degradation (see Table 2). Instead, the fixed residue at a high temperature, equal to 3% for the base polymer even after the mixing phase, increases to 12%: lower than the nominal content of basalt fibers. The latter data, probably caused by the poor representativeness of the tested sample compared to its bulk, could also be due to the thermal degradation of the organic constituents of the included filler. This last aspect was not explored in depth as it goes beyond the objectives of this research.

**Figure 2:**
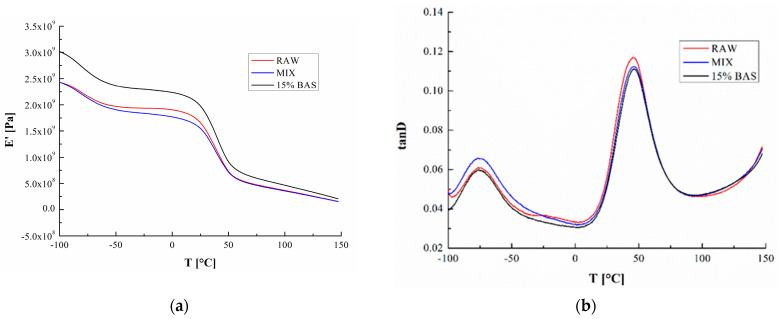
(**a**) Representative curves of storage modulus E’, (**b**) representative curves of tanD.

The joining point between thermal and mechanical characterization is represented by thermomechanical dynamic tests. The results provided by the DMTA are shown in Figure 2a,b, showing the variation in the storage modulus and the loss factor Tan δ as a function of temperature, respectively, for all the materials considered. The processing of these curves allowed us to obtain some characteristic parameters collected in Table 3. In particular, from Figure 2b, two signal (molecular transitions) can be noted. The phenomenon that is observed at lower temperatures, around −76 °C, represents a secondary transition related to the movements of small chain segments [30], while the primary (glassy) transition is centered at 46 °C. Evidently (see Table 3), the processing of the recovered PA12 and the compounding process do not influence these transitions, but the presence of the filler causes a significant increase in the storage modulus, showing a 26% increase at −100 °C and 19% increase at 25 °C with respect to the neat samples. This trend is rational since the existence of rigid structures in polymer composite materials induces the reduction in molecular chains’ mobility and produces a decrease in whole-material deformability. Furthermore, the results showed no significant effect on the glass transition temperature of the polymer matrix.

### 3.2. Mechanical Characterization

**Figure 3:**
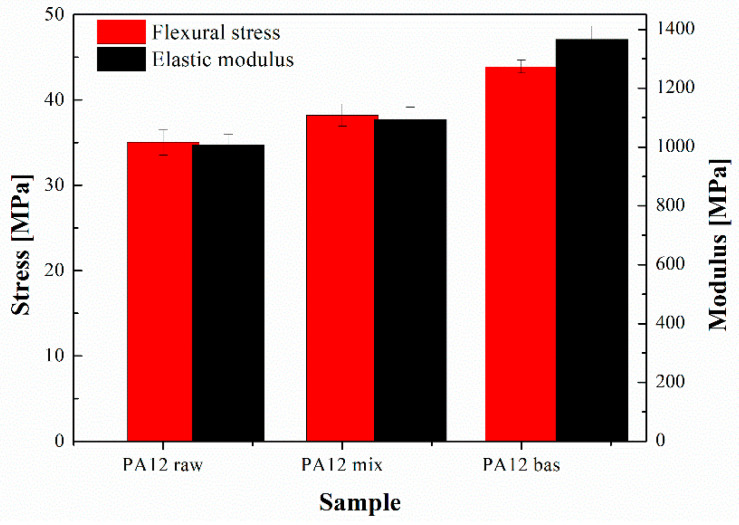
Results of 3-point bending tests.

In line with the previous considerations, it seems that the process slightly influences both the maximum flexural stress and the flexural elastic modulus of the neat matrix (see Figure 3 and Table 4). The addition of the inorganic filler causes a 40% increase in the flexural modulus of the composite (1366 MPa) compared to the pure PA12 raw and mix materials (1008 and 1094 MPa, respectively). Furthermore, the flexural strength (maximum stress) only slightly improved for the neat PA12 following the compounding step, which seems to undergo a further increase of about 16% (43.9 MPa) following the inclusion of basalt powder. These effects, partially expected for the addition of powders that are more rigid than the host polymer matrix, also generally reflect a quite good dispersion of the filler [31] as well as the nucleating ability of the basalt powder reported in [21,32].

### 3.3. Material Comparison

In order to obtain some useful concluding remarks, it is important to compare the material proposed in this study, PA12 with 15% basalt filler and the commercial alternatives, PA6 with 30% glass fibers and polypropylene with 30% glass fibers (Table 5). The results demonstrate that although, from a thermal point of view, the characteristics of PA12 are quite similar to those of PA6 and superior to those of polypropylene, the mechanical properties are still far from those of the competition, but the inclusion of basalt powders can compensate for this drawback. Additionally, PA12 is 25% lighter than PA6 and only 10% heavier than polypropylene. Another important aspect is represented by the material’s price. The PA12 scraps result in 2300% more money waste than PA6 and 360% more than polypropylene, and their recovery and reuse process is crucial. Finally, it is noteworthy that PA12 is the only fully biobased polymer contained in Table 5; there also exist biobased alternatives for PA6 and polypropylene, but their characteristics are sensitively worse than the petroleum-based commercial version.

## 4. Conclusions

Summarizing, due to its thermal and mechanical process, the PA12 polymer that was recovered from not-sintered SLS powders and reinforced with 15 wt% of mineral-based basalt powder allowed us to obtain, by compression molding, a material which could be properly employed in the automotive sector for interior, cover and shell manufacturing.

This research work can also be useful for many future detailed studies. First of all, it should be useful to test other compounds by increasing the basalt filler content, thus trying to further improve mechanical properties and reach a stiffness similar to that of polypropylene. Increasing the amount of filler caused an increase in composite density in comparison to that of the neat polymeric sample. A negligible difference between the calculated and measured density values is found in the literature from the small amount of voids and pores in composite structures. The void volume content indicates that the structure of composites containing up to 20 wt% of the filler is almost free of defects, resulting from sample porosity, and in [33], composites containing a <1% fraction of voids exhibit excellent quality. From a technological point of view, the possibility of involving other manufacturing processes can also be taken into account, for instance, FDM, even if further heating steps which could produce excessive thermal degradation should be avoided, or injection molding, having the objective to improve workpiece quality and material compaction. All these choices, as well as the best compression molding procedure, must be guided by a sustainability study, and therefore, it is also very important to perform a complete LCA. This analysis would allow us to quantify the reduction in the impact obtained through the recovery procedure, identify the factors that contribute the most to the reduction in the impact and identify optimal strategies to ensure the least waste of energy and resources.

## Figures and Tables

**Table 1:** DSC results.

	PA12 Raw	PA12 Mix	PA12 + 15% Basalt
T melting 1st [°C]	185	175	175
T melting 2nd [°C]	173	169–175	168–175
T crystallization [°C]	142	150	148
χ [%]	34.5	43.5	45.7

**Table 2:** TGA results.

	PA12 Raw	PA12 Mix	PA12 + 15% Basalt
T onset [°C] (2% loss)	339	348	361
T peak [°C]	459	448	458
Residue [%]	3	3	12

**Table 3:** DMTA results.

	PA12 Raw	PA12 Mix	PA12 + 15% Basalt
Storage modulus (−100 °C) [MPa]	2406 ± 117	2434 ± 134	3025 ± 151
Storage modulus (25 °C) [MPa]	1656 ± 42	1541 ± 59	1974 ± 95
Tβ [°C]	−76	−75	−76
Tg [°C]	45	46	46

**Table 4:** Three-point bending test results.

	PA12 Raw	PA12 Mix	PA12 + 15% Basalt
Maximum stress [MPa]	35.03 ± 1.51	38.22 ± 1.30	43.91 ± 0.77
Elastic modulus [MPa]	1008 ± 36	1094 ± 42	1366 ± 45

**Table 5:** Comparison of PA12 + 15% basalt and other commercial reinforced polymeric materials.

	PA6	Polypropylene	PA12
Commercial name	Akulon Ultraflow^®^ K FG6	PETRONAS Propelinas H022	SINTERIT^®^ (Kraków, Poland) PA12 Industrial FRESH
Reinforcement	30% glass fibers	30% glass fibers	15% basalt powder
Flexural modulus [MPa]	8500	3100	1366
Flexural strength [MPa]	235	47	44
Tg [°C]	55	−20	55
Tm [°C]	220	160	180
Density [g/cm^3^]	1.35	0.91	1.01
Price [EUR/kg]	5.00	26.00	120.00

## Data Availability

The raw data supporting the conclusions of this article will be made available by the authors on request.
